# Repeated Bereavement Takes Its Toll on Subjective Well-Being

**DOI:** 10.1093/geroni/igz047

**Published:** 2019-12-21

**Authors:** Frank J Infurna, Axel Mayer

**Affiliations:** 1 Department of Psychology, Arizona State University, Tempe; 2 Department of Psychology, RWTH Aachen University, Germany

**Keywords:** Adult life-span development, Household Income and Labour Dynamics of Australia Study (HILDA), Multilevel modeling, Reaction and adaptation to life events, Repeated adversity

## Abstract

**Background and Objectives:**

The bereavement literature has shown that losing close loved ones can lead to sustained declines in quality of life. Research in this area has typically focused on singular bereavement events, such as the loss of a spouse or child. Much less is known regarding the consequences of repeated bereavement or repeated losses in one’s social network.

**Research Design and Methods:**

We use longitudinal panel survey data from the Household Income and Labour Dynamics of Australia study to examine the effect of repeated bereavement in one’s social network on cognitive and affective measures of subjective well-being and whether there are age differences in the magnitude of these effects across young adulthood, midlife, and old age. To address our research questions, we use a multiple-group discontinuous change model with random effects.

**Results:**

Repeated deaths in one’s social network had a nonlinear effect on life satisfaction and positive affect, suggesting that individuals were able to adapt to two bereavements, but each bereavement beyond two resulted in sustained lower levels. Negative affect did not show increases because of repeated bereavements. Repeated bereavement had the strongest effect for those in young adulthood and old age.

**Discussion and Implications:**

Our findings demonstrate that repeated bereavement has consequences for subjective well-being and that young and older adults are most vulnerable to repeated bereavement. Our discussion focuses on the conceptual and methodological advancements of our study for the examination of major life stressors.

**Translational Significance**

Repeated losses in one’s social network can have detrimental consequences for subjective well-being. Individuals in young adulthood and old age are most vulnerable to repeated losses in one’s social network.

## Repeated Bereavement Takes its Toll on Subjective Well-Being

Throughout the course of one’s life, major life events, both positive and negative, have the potential to shape the course of development (Baltes, 1987; Birren & Cunningham, 1985; Hultsch & Plemons, 1979). The literature is storied in this regard with previous research documenting how spousal loss, unemployment and onset of chronic illness, among other life events, lead to sustained changes (declines) across domains of psychological well-being (Diener, Lucas, & Scollon, 2006; Infurna & Luthar, 2016; Lucas, 2007). For example, a meta-analysis by Luhmann, Hofmann, Eid, and Lucas (2012) found that spousal loss, divorce, and unemployment resulted in sustained declines across measures of cognitive-evaluative and affective well-being, whereas marriage and childbirth resulted in increases in well-being that were not sustained over time. This literature has numerous strengths, but usually focuses on single events and not on the role of repeated events that transpire over several years or decades and their cumulative consequences for development across the adult life span (for notable exception, see Luhmann & Eid, 2009). The examination of repeated life events is typically the focus of daily-diary research designs (Almeida, 2005; Schilling & Diehl, 2014). In this article, we bring together the major life events and daily stressor traditions by investigating the effects of repeated losses of social network members on subjective well-being in young adulthood, midlife, and old age. We use 15-year longitudinal panel survey data from the Household Income Labour Dynamics of Australia Study to examine whether repeated bereavement in one’s social network is associated with declines in subjective well-being (life satisfaction, positive and negative affect) and whether there are age differences across those in young adulthood, midlife, and old age in such changes.

## The Impact of Major Life Events on Development

The study of the impact of major life events dates to Holmes and Rahe’s (1967) seminal article that examined how various life events and their severity influence mental health and well-being. In life-span developmental psychology, it has long been postulated that (negative and positive) life events have the potential to shape the course of development, with there being differentiation among pathology, mortality, and non-normative event processes (see Baltes & Nesselroade, 1979; Diehl, 1999; Gerstorf & Ram, 2013; Hultsch & Plemons, 1979). The dominant approach in this regard has been to focus on single major life events and examine their potential influence on changes in mental health or subjective well-being. Lucas’ (2007) research has been instrumental in documenting that individuals are able to adapt to certain events, such as marriage and divorce, whereas the occurrence of unemployment, spousal loss, and disability lead to sustained declines in life satisfaction (see also, Infurna & Luthar, 2016, 2018).

An area of the literature that is much less developed is the role of *repeated* events over several years (and decades) on developmental change across pertinent domains of psychological functioning. There are various events that if experienced once, their impact may not be as immediate, whereas if they repeatedly occur, this has the potential to have long-term consequences for development (Luhmann, Orth, Specht, Kandler, & Lucas, 2014). One approach is to utilize a count measure of the number of adversities individuals have encountered over the course of their life, typically referred to as a cumulative life adversity score (Seery, 2011). Seery, Holman, and Silver (2010) have used this approach and observed an inverse U-shaped association in that people with some lifetime adversity reported better mental health and well-being than people with a high history of adversity and those with no history of adversity. Individuals with moderate levels of lifetime adversity were also found to report better physiological reactivity to laboratory stressors (Seery, Leo, Lupien, Kondrak, & Almonte, 2013). This approach uses the cumulative life adversity score as a *between-person difference* variable and provides meaningful insights by comparing persons that experienced different levels of adversity showing that adversity experienced over the course of one’s lifetime can have some potential benefits (for discussion, see Infurna & Jayawickreme, 2019; Jayawickreme & Blackie, 2014). This is in contrast to our interest in the role of repeated life events on *within-person* changes over one’s lifetime.

The leading study in the examination of repeated life events on developmental change is that of Luhmann and Eid (2009) who examined the consequences of repeated unemployment, divorce, and marriage on life satisfaction. Luhmann and Eid (2009) found that repeated unemployment led to sustained declines in life satisfaction (sensitization) and that this effect was stronger in men versus women. Focusing on repeated divorces, life satisfaction was higher at the second divorce than it had been at the first divorce; this is indicative of adaptation. Life satisfaction did not show differences between the first and second marriage. The study by Luhmann and Eid (2009) holds numerous strengths, including long course examination of repeated major life events and showing that there is a great deal of interindividual heterogeneity in the effects of repeated events and that the effects differ by the specific event considered. It is important to also emphasize that Luhmann and Eid (2009) examined the impact of unemployment, divorce, and marriage sequentially over time, which is differentiated from the cumulative lifetime adversity score.

### Multidimensional Approach

Life-span development is multidimensional in nature in that domains of functioning are comprised of multiple facets that are related (Baltes, 1987), and have the potential to show differential patterns of change over the life span and in relation to major life events (Infurna & Luthar, 2017a). This was recently shown in the adult resilience literature with facets of subjective well-being and physical health exhibiting different trajectories before and after spousal loss and child loss (Infurna & Luthar, 2017a, 2017b). For example, positive and negative affect showed quicker recovery to spousal and child loss, as compared to life satisfaction. Luhmann and colleagues (2012) also established this in their meta-analysis where they observed that cognitive-evaluative well-being shows stronger declines (changes) following major life events compared to affective well-being. Previous research that has examined the effect of repeated life events on development has solely focused on changes in life satisfaction (Luhmann & Eid, 2009). We extend this by additionally focusing on positive and negative affect. Although interrelated, based on previous research (see Anusic, Yap, & Lucas, 2014; Infurna & Luthar, 2017a, 2017b), they may show differential patterns of change. Individuals may show more sustained declines in life satisfaction over time, whereas they may be quicker to adapt to changes in affective well-being.

## Repeated Bereavement in Social Networks

Why focus on repeated deaths in one’s social network? Researchers have long documented the benefits of different components of social networks for health and well-being. Early research found that individuals who were more integrated in their network had a reduced likelihood of chronic illness, disability, and mortality (Berkman, Glass, Brissette, & Seeman, 2000; House, Landis, & Umberson, 1988). This has continued to be observed (Holt-Lunstad, Smith, Baker, Harris, & Stephenson, 2015), along with the ability of social networks in promoting resilience to and coping with adversity and stress (Cohen & Wills, 1985; Luthar et al., 2000; Zautra, Hall, & Murray, 2008). When examining the consequences of losses of social network members, most of the literature has focused on spousal, parental, or child bereavement. This research has been instrumental in showing that the loss of a spouse, parent, or child can lead to sustained changes across pertinent domains of functioning (Floyd, Mailick Seltzer, Greenberg, & Song, 2013; Infurna & Luthar, 2017a, 2017b; Stroebe & Schut, 2010). We utilize a repeated events approach to examine whether and how repeated deaths in one’s social network (i.e., close friends and family members) impacts changes in facets of subjective well-being (life satisfaction, positive and negative affect) over time.

The vast heterogeneity in the effects of bereavement on pertinent outcomes has been well-documented (Infurna & Luthar, 2018; Stroebe & Schut, 2010). This entails that the extent to which individuals are able to adapt and adjust to the loss of their loved one greatly differs across individuals. The death of a loved one may lead to sustained declines in various pertinent domains, such as well-being and health (Stroebe & Schut, 2010), as well as in some situations come as a relief after long periods of suffering and intensive caregiving (Schulz et al., 2003). Furthermore, the closeness between the survivor and decedent, the cause of death, and circumstances surrounding their death play an instrumental role in one’s ability to adapt to the bereavement (Boerner & Heckhausen, 2003; Carr, 2003; Fry, 2001). The impact of the loss is not confined to the time surrounding death, but potential secondary stressors that arise. Readjustment following bereavement may involve a complexity of changes, amongst other things, changes in daily routine, relocation, changes in the nature and function of relationships with social network members (e.g., no longer having a close confidant to go to in times of need for emotional and instrumental support), financial resources, physical health, and the potential future one envisioned with their loved one (Antonucci, Akiyama, & Merline, 2001; Fry, 2001). These secondary stressors of bereavement can have downstream consequences for subjective well-being both over the short- and long-term.

One of the most prominent conceptual models of social networks and life-span development is the Convoy Model proposed by Kahn and Antonucci (1980). The Convoy Model utilizes a life-span perspective upon which to conceptualize the nature of social relations, their meaning, structure, and development across the adult life span (Antonucci, 1994). The convoy consists of the support relations experienced by the individual. Thereby, the convoy represents the composition of one’s social network, which is shaped by personal (i.e., age, gender) and situational (i.e., role expectations, resources, demands) characteristics. A key component of the Convoy Model that is most pertinent for the current investigation is that one’s social network composition is dynamic and constantly changing and evolving across the life span. As individuals change and develop across the life span from young adulthood to old age, so too does their social network composition and the quality of their relationships (see Antonucci et al., 2001). Depending on one’s stage in the life span, their social network composition can drastically differ. For example, in midlife, one’s social network composition is likely to be comprised of family members (i.e., spouse/partner, parents, siblings), as well as close friends and periphery friends (i.e., coworkers; Antonucci et al., 2001). Conversely, in old age, through selective pruning and changes in work status (i.e., retirement), one’s social network size is reduced and may primarily consist of close family members and friends whose time in the network has been longer and survived the test of time (Antonucci, 2001). The Convoy Model postulates that the processes of changes in social network composition are an *active* and *dynamic* process, whereas losses in one’s social network as a function of repeated bereavement is a process that the individual *is not* actively choosing and goes against the expectations of the Convoy Model. Repeated bereavement in one’s social network through the loss of family members and close friends is largely outside of one’s control and as a result, could lead to (sustained) declines in subjective well-being.

### Potential Courses of Change Following Repeated Bereavement

**Figure 1.**
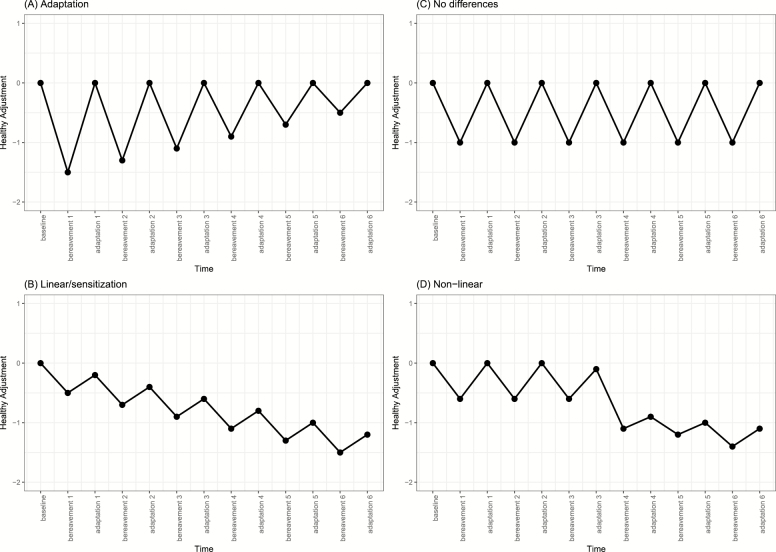
Possible changes in healthy adjustment outcomes as a function of repeated bereavement. Figure 1A illustrates adaptation: Individuals are able to adapt with each bereavement leading to less strong declines and levels returning to baseline after each repeated bereavement. Figure 1B illustrates linear/sensitization: Each bereavement is associated stronger and sustained declines. Figure 1C illustrates no differences in repeated bereavements: Individuals show similar changes and adaptation with each bereavement. Figure 1D illustrates nonlinear effects of repeated bereavements: Individuals are able to recover/adapt to the first several bereavements, but reach a threshold and show more substantial and sustained declines once the threshold is met.

Luhmann and Eid (2009) discuss different patterns through which individuals’ well-being changes because of repeated events. In this study, we specifically target multiple instances of reaction (change at the time of bereavement) and adaptation (one’s ability to bounce back and have well-being return to previous levels) following repeated bereavement. Figure 1 graphically illustrates four possible scenarios for how well-being changes because of repeated bereavement. Individuals may adapt to repeated events by levels of well-being always returning back to baseline following the event and magnitude of the decrease in well-being being less with each successive event (Figure 1A). A second scenario is that of sensitization, where with each experience of the event, well-being continues to decrease (Figure 1B). Third, there could be no differences in the magnitude of reaction and adaptation to repeated bereavement with individuals showing the same consequences and adaptation with each bereavement (Figure 1C). A fourth scenario is that of a nonlinear impact; Individuals are able to show relatively quick adaption to the first and second bereavements, but repeated bereavement above three (or more) takes its toll, leading to stronger and sustained declines in well-being (Figure 1D). Previous research has provided evidence for each of these scenarios in different contexts, except nonlinear consequences (see Luhmann & Eid, 2009).

### Age Differences in Repeated Bereavement

The developmental stage in adulthood has the potential to influence the consequences of repeated bereavement in one’s social network on subjective well-being. As discussed previously, the Convoy Model asserts that social networks are dynamic and evolving over the life span. Socioemotional selectivity theory (SST; Carstensen, Isaacowitz, & Charles, 1999) additionally focuses on social network composition changes across the adult life span and their functionality for development. SST has long postulated that there is a selective reduction in the amount of social interaction that begins as early as in young adulthood, whereas emotional closeness to significant others increases during adulthood and into old age (Carstensen, 1992; Carstensen et al., 1999). Especially in old age, this selective pruning of social network members is paramount, where the emphasis shifts from having a larger number of friends in young adulthood to reductions in midlife and in old age. Despite changes in social network composition and size, the closeness or strength of social relationships remains stable, even increasing in older adults.

Depending on one’s stage in the life span, there are different goals, activities, and composition of the network that individuals are engaging in. In young adulthood and midlife, social networks are larger and more dynamic due to numerous roles (e.g., work, family), resulting in possible better adaptation (Antonucci et al., 2001; Lachman, 2004). However, the deaths of peers may be off-time and unexpected, leading to more detrimental consequences, whereas among those in old age, deaths may be timely and anticipated, which could potentially condition their psychological effects. In old age, individuals may be less likely to fill the void left by the loss of social network members. We expect that repeated bereavement would result in stronger and more sustained declines in well-being in older adults, compared to those in young adulthood and midlife. As discussed by SST, the development of one’s social network in old age is an active process of selecting those who best fulfill one’s emotional goals (see also, Blanchard-Fields, 2007) and a loss can be of greater magnitude for older adults due to a larger void and difficulty in filling the void left by the death of a close family member or friend.

## The Present Study

The major objectives of the present study are to examine the nature of repeated bereavement on multiple facets of subjective well-being across the adult life span. First, we examine whether repeated bereavement of family members and close friends leads to sustained changes in life satisfaction, and positive and negative affect. A repeated events approach is important for this focus on family members and close friends because losing network members leads to structural changes in the composition of one’s social network. Given the novel nature of our approach and study, it is largely unexplored which of the four patterns of change individuals will follow as depicted in Figure 1. Based on the conceptual features of the Convoy Model, we hypothesize that repeated bereavement will have a nonlinear effect as documented in Figure 1D in that individuals, on average, will have the ability to adapt to the first bereavements, but with each successive bereavement beyond a threshold, this will lead to stronger declines in subjective well-being. Throughout the course of adulthood, social network composition is dynamic and evolving and changes that transpire due to bereavement can be detrimental to one’s subjective well-being. Second, we examine whether repeated bereavement has a differential effect across individuals who are in young adulthood, midlife, and old age. We hypothesize that older adults will be more strongly affected by repeated bereavement (sensitization). As discussed above, SST postulates that social relationships and network composition becomes more selective in older ages, which assumes that each social relationship forms a specific function; bereavements can therefore be especially detrimental for those in old age where network composition may show less plasticity.

## Method

We examined our research questions using data from 15 annual waves (2001–2015) of the Household Income and Labour Dynamics of Australia Study (HILDA). Comprehensive information about the design, participants, variables, and assessment procedures in the study are reported in Dyrenforth, Kashy, Donnellan, and Lucas (2010) and Watson (2010). A brief overview of details relevant to the present study is given below.

### Participants and Procedure

The HILDA is a nationally representative annual panel study of private households and their inhabitants initiated in 2001 that includes residents of Australia. Within a household, all persons aged 15 years and older were invited to participate. Data are collected annually via a combination of face-to-face and telephone interviews and self-completed questionnaires.

For the present study, we analyzed data from the 4,081 participants who reported that they lost at least three family members or close friends over the course of the study. To maximize the longitudinal assessments available for the time-series, we only included participants from the original cohort assessed in 2001. Participants were, on average, 50.46 years of age (*SD* = 15.48, range 15–93) at the baseline assessment, and, on average, 51.31 years of age (*SD* = 15.72, range 16–94) at the age of the first bereavement, and 57% were women. Education level is represented across eleven categories in the HILDA data set, ranging from less than high school to postgrad-masters or doctorate, and 62% attained at least a high school education.

### Measures

#### Bereavement in social network

At each wave, participants were asked whether during the past 12 months they experienced the “death of a close friend” and “death of other close relative/family member (e.g., parent or sibling)”. These two categories of bereavement are separately asked in the questionnaire. We selected participants who reported a bereavement across family members or close friends at three different waves of assessment during the course of their participation in the HILDA and we analyzed data for up to six bereavements over the course of 15 years of assessment. Of the 4,081 participants included in the present study, 1,166 (29%) experienced three bereavements across the two categories, 989 (24%) experienced four bereavements across the two categories, 697 (17%) experienced five bereavements across the two categories, and 1,229 (30%) experienced six or more bereavements across the two categories.

#### Outcomes

*Life satisfaction* was reported on annually, answering the question “How satisfied are you with your life, all things considered?” using a 0 (*totally unsatisfied*) to 10 (*totally satisfied*) rating scale. This item has been widely used in psychological research (see Cheung & Lucas, 2014; Gerstorf et al., 2008). On average, participants contributed 14.13 observations (range 4–15).

*Positive and negative affect* were assessed at each wave using questions starting with the stem “How much of the time during the past 4 weeks …” and answered on a scale from 1 (*all of the time*) to 6 (*none of the time*) (see Anusic et al., 2014). *Negative affect* items were “Have you been a nervous person?,” “Have you felt so down in the dumps nothing could cheer you up?,” Have you felt down?,” “Did you feel worn out?,” and “Did you feel tired?”. *Positive affect* items were “Did you feel full of life?,” “Have you felt calm and peaceful?,” “Did you have a lot of energy?,” and “Have you been a happy person?”. Items for each were averaged with higher scores for each indicating more frequent experience of affect. Cronbach’s α ranged from .80 to .85 at each wave for negative affect and from .81 to .86 at each wave for positive affect. On average, participants contributed 13.55 negative affect observations (range 3–15) and 13.54 positive affect observations (range 3–15).

### Statistical Procedures

Data were analyzed using multilevel models with random slopes (Grimm, Ram, & Estabrook, 2017; Singer & Willett, 2003). We used a discontinuous change model that is adapted from the approach used by Luhmann and Eid (2009) in their study examining repeated unemployment, marriage, and divorce (see also Lucas, Clark, Georgellis, & Diener, 2003). In doing so, we created time-varying binary indicator variables for reaction and adaptation that constitute the assessment when the bereavement occurred (reaction) and the period after the event onset (adaptation) until the next event. We also created a binary indicator for the baseline assessment that constitutes the period before the first bereavement event. In our model, we included the baseline indicator as well as all reaction and adaptation variables, except for the first reaction variable that serves as the reference. In building our model, we first estimated random slopes for all indicator variables in the model. In testing the model with all of the random slopes for reaction and adaptation, we encountered that the variances of the random slopes for the reaction indicators were numerically close to zero and nonsignificant, indicating that there were no between-person differences in reaction to each bereavement. Since the Bayesian Information Criterion (BIC) also preferred the model without random slopes for reaction variables, we decided to omit these random slopes and solely included fixed effects for reaction variables in subsequent analyses. The random slopes were estimated for the adaptation variables, indicating that individuals potentially differed in the extent to which their subjective well-being bounced back following bereavement, but there were no between-person differences in the extent to which bereavement was linked to immediate changes in subjective well-being (i.e., reaction). The level 1 equation for an individual *i* at occasion *t* in our basic model thus is given by:

$${\text{Y}}_{it}=\beta_{0i}+\beta_{1i}({\text{Baseline}}_{it})+\beta_{2i}({\text{Adaption1}}_{it})+\beta_{3}({\text{Reaction2}}_{it})+\beta_{4i}({\text{Adaption2}}_{it})+...+\beta_{11}(Reaction6_{it})+\beta_{12}i(Adaptation6_{it})+e_{it}$$

Where Y_*it*_ refers to the outcome variable considered (life satisfaction, positive or negative affect) and Baseline, Reaction, Adaption refer to the indicator variables as described above. Our analyses included individuals who reported at least three bereavements and up to six bereavements. β _0*i*_ refers to individuals’ levels in each outcome at the time of the first reported bereavement; β _1*i*_ (Baseline_*it*_) contrasts levels of the outcome at the time period before the first bereavement to levels of the outcome at the reference (i.e., at the first bereavement); β _2*i*_ (Adaption1_*it*_) and each adaptation parameter refers to the amount of difference in the outcome during the adaptation phase compared to the time of the first reported bereavement; β _3_ (Reaction2_*it*_) and each reaction parameter refers to the amount of difference in the outcome at the corresponding bereavement compared to the time of the first reported bereavement. Notice that the β coefficients for the reaction parameters do not have a subscript *i*, indicating that these do not vary across individuals. In referring to the reaction parameters, if the parameter is negative and significant, this indicates that levels are lower at that bereavement in relation to levels at the first bereavement, suggesting that there is a substantially lower level. If the reaction parameter is not significant, this suggests that individuals do not show sustained changes in the outcome. In referring to the adaptation parameters, if the parameter is negative and significant, this indicates that levels are lower at that assessment period in relation to levels at the first bereavement, suggesting that there is no adaptation. If the adaptation parameter is not significant, this suggests that individuals are able to bounce back and return to previous levels in the outcome.

Our second research question focuses on whether there are age-differences in the effect of repeated bereavement on subjective well-being. To examine age differences, we further extended Luhmann and Eid`s (2009) approach by simultaneously fitting the model described above in three age groups (young adulthood: 15–39 years of age at Time 1, middle age: 40–59 years of age at Time 1, and old age: 60 years old of age and older at Time 1) using a multi-group structural equation modeling approach with random slopes. The multi-group approach enabled us to test for age group differences in parameter estimates using a Wald Test. The Wald Test is computed based on an unrestricted model and tests if the fixed effects of the reaction and adaptation indicator variables in our discontinuous change model are the same across the three age groups. If the Wald Test is significant, then this indicates that we cannot assume the reaction and adaptation fixed effects are the same, but differ across the three age groups. If the Wald Test is not significant, this indicates the reaction and adaptation fixed effects are the same across the three age groups.

The models were estimated with a robust maximum likelihood estimator in Mplus 7.2 (Muthén & Muthén, 2012) using a two-level multi-group specification with random slopes.

## Results

Our results are divided into two sections. First, we present models that examine the overall effect of repeated bereavement on subjective well-being. Second, we examine whether there are age differences in the extent to which repeated bereavement affects subjective well-being.

### Repeated Bereavement in Social Networks

Table 1 shows the results from our models examining the effects of repeated bereavement on life satisfaction, positive affect, and negative affect. Focusing on life satisfaction, there was, on average, an initial decline with the onset of the first bereavement (estimate = 0.07, *p* < .05); this indicates that life satisfaction was, on average, .07 points higher at baseline. Following the first bereavement, there was relative stability with the second and third bereavements as indicated by the nonsignificant bereavement and adaption parameters. However, the fourth (estimate = −0.05, *p* < .05), fifth (estimate = −0.07, *p* < .05), and sixth (estimate = −0.12, *p* < .05) bereavements resulted in significant declines in life satisfaction, relative to levels at the first bereavement. Focusing on adaptation, on average, individuals were able to bounce back as indicated by the adaptation parameters being nonsignificant, except for that of adaptation to the sixth bereavement (estimate = −0.10, *p* < .05).

**Table 1.** Examining the Effects of Repeated Bereavement on Life Satisfaction, Positive Affect, and Negative Affect

	Life satisfaction	Positive affect	Negative affect
	Estimate (*SE*)	Estimate (*SE*)	Estimate (*SE*)
Fixed effects			
Intercept at Bereavement 1	7.96* (0.02)	3.99* (0.02)	2.33* (0.01)
Baseline	0.07* (0.02)	0.09* (0.01)	0.002 (0.01)
Adaptation 1	−0.002 (0.02)	0.09* (0.01)	−0.03* (0.01)
Bereavement 2	0.01 (0.02)	−0.04* (0.01)	−0.001 (0.01)
Adaptation 2	−0.01 (0.02)	0.01 (0.01)	−0.04* (0.01)
Bereavement 3	−0.02 (0.02)	−0.08* (0.01)	0.01 (0.01)
Adaptation 3	−0.01 (0.02)	−0.03* (0.01)	−0.05* (0.01)
Bereavement 4	−0.05* (0.02)	−0.09* (0.01)	0.01 (0.01)
Adaptation 4	0.02 (0.02)	−0.05* (0.01)	−0.03* (0.01)
Bereavement 5	−0.07* (0.03)	−0.10* (0.02)	0.02 (0.02)
Adaptation 5	−0.01 (0.03)	−0.09* (0.02)	−0.01 (0.02)
Bereavement 6	−0.12* (0.04)	−0.11* (0.02)	0.02 (0.02)
Adaptation 6	−0.10* (0.03)	−0.19* (0.02)	0.01 (0.02)
Random effects			
Intercept	1.09* (0.04)	0.64* (0.01)	0.46* (0.01)
Baseline	0.46* (0.04)	0.14* (0.01)	0.09* (0.01)
Adaptation 1	0.12* (0.03)	0.05* (0.01)	0.03* (0.01)
Adaptation 2	0.09* (0.03)	0.02* (0.01)	0.01 (0.01)
Adaptation 3	0.14* (0.03)	0.06* (0.01)	0.04* (0.01)
Adaptation 4	0.24* (0.05)	0.12* (0.02)	0.09* (0.02)
Adaptation 5	0.39* (0.11)	0.10* (0.02)	0.07* (0.02)
Adaptation 6	0.24* (0.05)	0.12* (0.02)	0.09* (0.01)
Residual variance	1.01* (0.02)	0.37* (0.01)	0.27* (0.01)
Model Fit Statistics			
Bayesian Information Criterion	181,255	119,862	102,620

*Note*: *N* = 4,081.

*p < .05.

Each bereavement resulted in declines in positive affect as compared to levels of positive affect at the first bereavement. Losing a social network member as a result of death was associated with consistent declines in positive affect (estimates for bereavements 2 through 6 are all negative and significant). Adaptation shows a different pattern to that of reaction; during the adaptation periods following the first two bereavements, individuals, on average, were able to bounce back to previous levels, whereas in the adaptation periods following the third through six bereavements, individuals showed sustained declines in positive affect. This is signified by the adaptation parameters being significant and negative, suggesting that positive affect levels remained lower than at the first bereavement, following the third through sixth bereavements.

Negative affect showed little changes in response to repeated bereavements over the course of the study. The only significant parameters are that of adaptation to the first through fourth bereavements. On average, negative affect bounced back or improved following the first four bereavements.

Across the three outcomes, there were between-person differences in the extent to which individuals were able to bounce back following each bereavement. This is suggested from the random effects variances for each of the adaptation variables being significant in the bottom of Table 1. In the next sets of analyses, we used multiple-group models to examine whether there were age differences in the extent to which repeated bereavement affected each outcome.

### Age Differences in Repeated Bereavement

In a next step, we conducted a multiple-group model that included three groups based on age at Time 1: young adulthood (15–39), midlife (40–59), and old age (age 60 years and older). The results from this model are shown in Table 2. To determine whether there were significant age differences in reaction and adaptation to repeated bereavements we used a Wald Test (see section Statistical Procedures above). For each outcome, the Wald Test is significant, indicating that there are differences in the extent to which individuals react and adapt to repeated bereavements in young adulthood, midlife, and old age.

**Table 2.** Examining the Effects of Repeated Bereavement on Life Satisfaction, Positive Affect, and Negative Affect: Separately in Young Adulthood, Midlife, and Old Age

	Life satisfaction			Positive affect			Negative affect		
	Young adulthood	Midlife	Old age	Young adulthood	Midlife	Old age	Young adulthood	Midlife	Old age
	Estimate (*SE*)	Estimate (*SE*)	Estimate (*SE*)	Estimate (*SE*)	Estimate (*SE*)	Estimate (*SE*)	Estimate (SE)	Estimate (*SE*)	Estimate (*SE*)
Fixed effects									
Intercept at Bereavement 1	7.73* (0.04)	7.86* (0.03)	8.40* (0.04)	3.99* (0.03)	3.98* (0.02)	4.03* (0.03)	2.48* (0.02)	2.32* (0.02)	2.17* (0.02)
Baseline	0.12* (0.04)	0.002 (0.03)	0.14* (0.04)	0.14* (0.02)	0.04* (0.02)	0.10* (0.02)	−0.02 (0.02)	0.03* (0.01)	−0.03 (0.02)
Adaptation 1	−0.01 (0.04)	−0.01 (0.03)	0.04 (0.04)	0.06* (0.02)	−0.01 (0.02)	−0.02 (0.03)	−0.04 (0.02)	−0.02 (0.02)	−0.02 (0.02)
Bereavement 2	−0.04 (0.04)	0.04 (0.03)	0.03 (0.04)	−0.02 (0.03)	−0.04* (0.02)	−0.08* (0.02)	−0.02 (0.02)	0.01 (0.02)	0.01 (0.02)
Adaptation 2	−0.06 (0.04)	0.04 (0.03)	−0.03 (0.05)	−0.01 (0.03)	−0.01 (0.02)	−0.09* (0.03)	−0.09* (0.02)	−0.04* (0.02)	0.004 (0.02)
Bereavement 3	−0.09* (0.04)	0.03 (0.03)	−0.03 (0.04)	−0.04 (0.03)	−0.07* (0.02)	−0.13* (0.03)	−0.02 (0.02)	0.001 (0.02)	0.06* (0.02)
Adaptation 3	0.01 (0.04)	0.06 (0.03)	−0.16* (0.04)	−0.01 (0.02)	−0.03 (0.02)	−0.19* (0.03)	−0.08* (0.02)	−0.07* (0.02)	0.05* (0.02)
Bereavement 4	−0.10* (0.05)	0.01 (0.04	−0.09* (0.04)	−0.09* (0.03)	−0.04* (0.02)	−0.17* (0.03)	0.03 (0.03)	−0.02 (0.02)	0.05* (0.02)
Adaptation 4	−0.05 (0.04)	0.11* (0.04)	−0.07 (0.05)	−0.08* (0.03)	−0.05* (0.02)	−0.22* (0.03)	−0.05 (0.03)	−0.06* (0.02)	0.05* (0.02)
Bereavement 5	−0.04 (0.06)	−0.05 (0.05)	−0.12* (0.06)	−0.06 (0.04)	−0.08* (0.03)	−0.19* (0.03)	0.01 (0.04)	−0.01 (0.02)	0.08* (0.03)
Adaptation 5	−0.10 (0.06)	0.05 (0.05)	−0.05 (0.07)	−0.13* (0.04)	−0.02 (0.03)	−0.19* (0.04)	0.01 (0.04)	−0.06* (0.02)	0.07* (0.03)
Bereavement 6	−0.22* (0.10)	−0.07 (0.07)	−0.17* (0.06)	−0.08 (0.06)	−0.07* (0.03)	−0.18* (0.04)	0.03 (0.05)	0.02 (0.03)	0.05 (0.03)
Adaptation 6	−0.09 (0.08)	0.03 (0.05)	−0.28* (0.05)	−0.19* (0.05)	−0.10* (0.03)	−0.32* (0.03)	0.08 (0.05)	−0.05* (0.03)	0.08* (0.03)
Random effects									
Intercept	0.96* (0.06)	1.17* (0.06)	0.85* (0.06)	0.56* (0.02)	0.70* (0.02)	0.64* (0.03)	0.43* (0.02)	0.48* (0.02)	0.41* (0.02)
Baseline	0.55* (0.07)	0.47* (0.06)	0.27* (0.06)	0.14* (0.02)	0.14* (0.01)	0.13* (0.02)	0.09* (0.01)	0.09* (0.01)	0.07* (0.01)
Adaptation 1	0.10* (0.04)	0.14* (0.04)	0.08 (0.07)	0.05* (0.02)	0.05* (0.01)	0.04* (0.02)	0.04* (0.02)	0.02 (0.01)	0.02 (0.01)
Adaptation 2	0.13* (0.05)	0.04 (0.03)	0.12* (0.05)	0.01 (0.01)	0.02 (0.01)	0.01 (0.01)	0.02 (0.01)	0.002 (0.01)	0.02 (0.01)
Adaptation 3	0.13* (0.05)	0.14* (0.04)	0.14* (0.06)	0.06* (0.02)	0.05* (0.01)	0.06* (0.02)	0.05* (0.02)	0.02 (0.01)	0.06* (0.02)
Adaptation 4	0.22* (0.07)	0.28* (0.09)	0.16 (0.10)	0.12* (0.03)	0.11* (0.02)	0.11* (0.03)	0.10* (0.04)	0.10* (0.02)	0.05* (0.02)
Adaptation 5	0.43* (0.16)	0.30 (0.17)	0.48* (0.21)	0.13* (0.04)	0.07* (0.02)	0.09* (0.03)	0.10* (0.03)	0.05* (0.02)	0.06* (0.03)
Adaptation 6	0.38* (0.11)	0.17* (0.05)	0.25* (0.08)	0.13* (0.04)	0.14* (0.03)	0.10* (0.02)	0.16* (0.04)	0.09* (0.02)	0.05* (0.02)
Residual variance	1.02* (0.03)	1.00* (0.03)	1.02* (0.04)	0.41* (0.01)	0.35* (0.01)	0.34* (0.01)	0.33* (0.01)	0.27* (0.01)	0.22* (0.01)
Age differences	Estimate (df)			Estimate (df)			Estimate (df)		
Wald Test	98.80* (24)	98.80* (24)	98.80* (24)	121.30* (24)	121.30* (24)	121.30* (24)	86.13* (24)	86.13* (24)	86.13* (24)
Model Fit Statistics									
Bayesian Information Criterion	181,184	181,184	181,184	120,092	120,092	120,092	102,383	102,383	102,383

**Figure 2.**
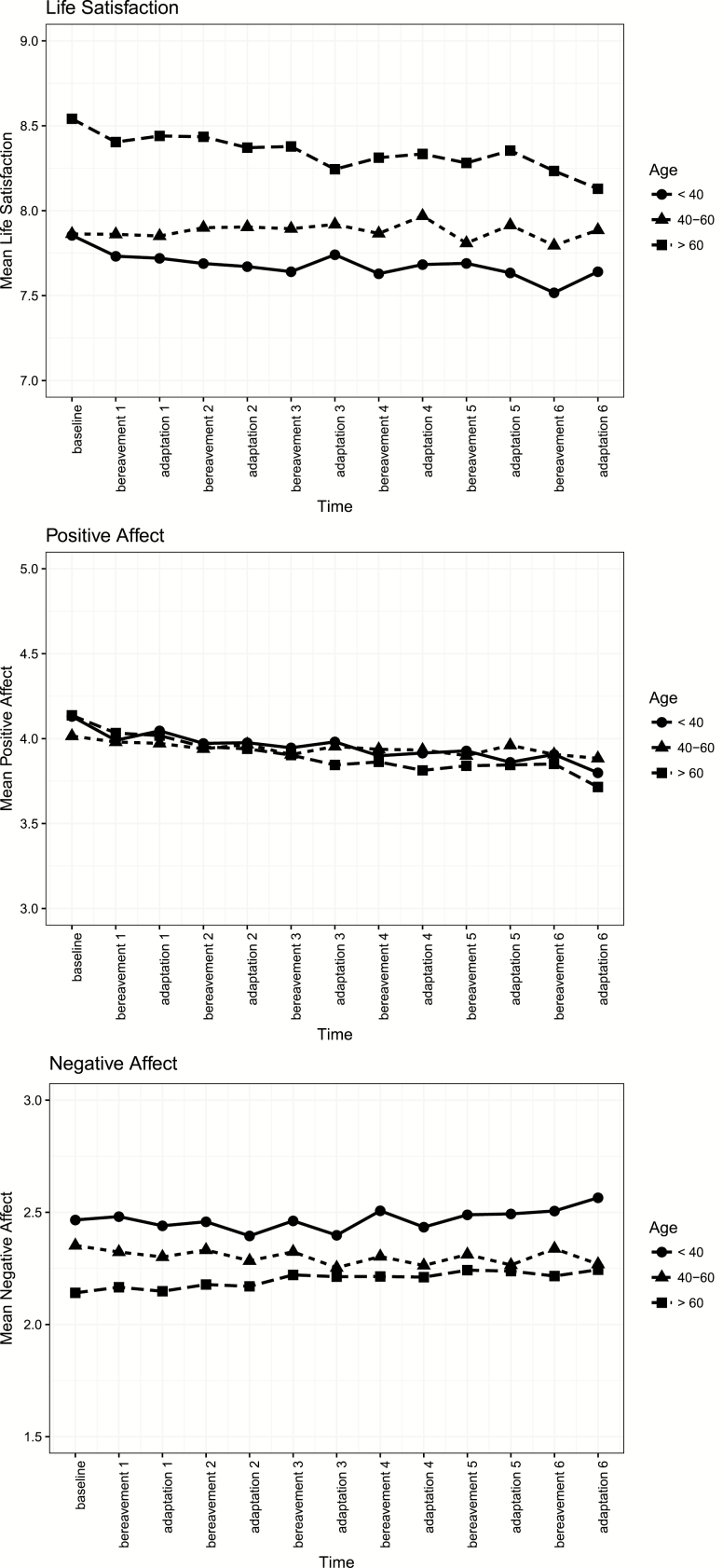
Model-implied changes in life satisfaction (A), positive affect (B), and negative affect (C) as a result of repeated bereavement in young adulthood (circle), midlife (triangle), and old age (square). We observed that young adults and older adults exhibited nonlinear effects of repeated bereavement on life satisfaction and positive affect, compared to individuals in midlife. For negative affect, there were little differences across the three age groups.

Figure 2A graphically illustrates the changes in life satisfaction because of repeated bereavements across the three age groups. There are clear level differences in life satisfaction across the three age groups, but more importantly, there are differences in the pattern of change in reaction and adaptation to repeated bereavement. Individuals in young adulthood and old age showed the strongest effects of repeated bereavement on life satisfaction. The effects of repeated bereavement were most profound beginning at bereavement three and four for the young adulthood and old age groups, indicating nonlinear effects of bereavement on life satisfaction.

Figure 2B graphically illustrates the changes in positive affect because of repeated bereavements across the three age groups. We did not observe level differences in positive affect across the three age groups. Individuals from across the adult life span show changes in positive affect because of repeated bereavement; the magnitude of these changes is strongest in midlife and old age. In young adulthood, the fourth, fifth and sixth bereavement parameters are statistically significant. In midlife, the second through six bereavement parameters are statistically significant, but the adaptation parameters are not, indicating that individuals were able to return to previous levels. In old age, each of the bereavement and adaptation parameters are negative and significant, suggesting that there are sustained declines in positive affect with each bereavement.

Figure 2C graphically illustrates changes in negative affect because of repeated bereavements across the three age groups. Individuals in old age, showed slight increases over time as a result of repeated bereavements and this effect was strongest in the later bereavements.

## Discussion

The major objective of the present study was to examine whether and how repeated bereavement is associated with changes in cognitive and affective measures of subjective well-being. We observed that repeated bereavement resulted in small declines in life satisfaction and positive affect, but little changes to negative affect. Taking a life-span approach, we examined whether the consequences of repeated bereavement differed across individuals in young adulthood, midlife, and old age. We found evidence for age differences in that repeated bereavement resulted in stronger declines in life satisfaction and positive affect for individuals in young adulthood and old age. Our findings demonstrate that repeated life stressors have the potential to shape the course of development and their effects differ across the adult life span. Our discussion focuses on the merits of examining the consequences of repeated life stressors and how this conceptual and methodological approach can be developed further.

### Repeated Bereavement in Social Networks

Repeated bereavement in one’s social network led to small changes across life satisfaction and positive affect, but not negative affect. This was found across the entire sample, which consisted of participants aged 15 to over 90 years of age. Changes in life satisfaction and positive affect resembled to what is illustrated in Figures 1B and 1C; there were slight declines with each successive bereavement and no differences in their magnitude and individuals, on average, being able to adapt in the time thereafter each repeated bereavement. Interestingly, negative affect did not follow the same pattern as life satisfaction and positive affect. This is in contrast to recent research showing negative affect consisting of more diverse types of trajectories or responses to spousal loss (Infurna & Luthar, 2017a) and in contrast to theoretical accounts of the set point theory because positive affect theoretically would seem most resilient to contextual influences. This further signifies how responses to bereavement are not uniform, but likely involve a great deal of heterogeneity. Our findings for life satisfaction and positive affect are in line with Luhmann and Eid’s (2009) findings on repeated marriage in that there were little differences between each event of marriage and differ from the findings of repeated unemployment leading to sustained declines in life satisfaction.

When a social network member dies, whether it be a spouse or child, close family member or friend, it has the potential to take a toll on the individual. Repeated bereavement means not only that an important source of one’s social support is gone, but one consistent consequence is that it leaves a void in the structure of one’s social network (Thoits, 1983). Social network members provide aid, affect, and affirmation—each being associated with better health and well-being for the individual (Antonucci, 2001; Berkman et al., 2000). Although close relationships are the most studied and shown to be the most pervasively important, there can be special circumstances under which a person generally not considered very close can have significant influence on the individual. Granovetter (1973) discusses how there is strength in weak ties, or periphery network members that individuals may not be in consistent contact with. A coworker or a friend who has the unique skills that are needed to solve a problem. Social network members serve a purpose and their loss, especially repeated, can result in a loss of available support that may not be able to be filled. Losses of this nature could be distressing because it triggers secondary stressors, such as strains and gaps in one’s social network that bear on subjective well-being.

Why did we observe such small effects of repeated bereavement in the entire sample? The magnitude of the changes are smaller to that of what has been previously observed for spousal loss and child loss (see Infurna & Luthar, 2017a, 2017b; Lucas et al., 2003; Luhmann et al., 2009). Losing a close friend or family member is not a trivial matter, but may not result in the same disruptions as losing a spouse or child, which could be the reason for the smaller effects. As we discussed above, repeated events of this nature can leave a void in one’s social network by having fewer people to interact with regularly, engage in hobbies and pleasurable activities with and go to in times of need. Although these network members are central, the effects may occur at a quicker time scale of weeks and months, with the initial effects (reaction) and adaptation occurring more quickly. Although the HILDA has the advantage of sampling a larger number of people on a yearly basis, permitting for such analyses, the yearly assessments maybe too sparse to detect immediate changes (reaction) and subsequent adaptation in subjective well-being. Future research examining repeated major life stressors will require more closely spaced observation to further examine immediate changes and further disentangle mechanisms underlying the effects, as well as studying indicators beyond that of well-being that could be more sensitive to changes following repeated bereavement (Infurna & Jayawickreme, 2019; Infurna & Luthar, 2018). We discuss this further below.

### Age Differences in Repeated Bereavement

The effect of repeated bereavement on subjective well-being differed among those in young adulthood, midlife, and old age. Individuals in young adulthood and old age showed the strongest declines in life satisfaction and positive affect because of repeated bereavement, compared to those in midlife. The effect of repeated bereavement on life satisfaction and positive affect in young adulthood and old age resembled that of Figure 1D, nonlinear effects; each repeated bereavement resulted in a decline, but the magnitude of the change and lack of adaptation increased with successive bereavements beginning with the third bereavement. The first two to three bereavements, individuals, on average, were able to bounce back and adapt, as indicated by the adaptation parameters for two and three being nonsignificant (this signifies that there were no differences in levels of life satisfaction and positive affect between these assessments and levels at the first bereavement). However, each successive bereavement thereafter was associated with stronger and sustained declines in life satisfaction and positive affect. It could be that individuals still have the network members in place or resources available to them to overcome some losses, but when a threshold is met, the void left by previous and current losses is too large to overcome. To combat losses in social roles and preserve their self-identity in the face of these deficits, individuals work to replace lost social roles with new, compensatory activities (Atchley, 1989; Cavan, Burgess, Havighurst, & Goldhamer, 1949). Although the loss of social network members may present an individual with changing expectations and social network composition, older adults will attempt to preserve continuity of attitudes dispositions, preferences, and behaviors throughout their life course. In the case of repeated bereavements, there is difficulty for older adults to combat losses in social roles through preservation of self-identity in the face of these deficits, working to replace lost social roles with new, and compensatory activities.

Our findings for older adults being more impacted by repeated bereavement is in line with conceptual models of social network composition and function across the adult life span. The Social Convoy Model and SST propose that changes in social network composition, make-up, and functionality across the adult life span are a dynamic, lifelong process (Antonucci, 2001; Carstensen et al., 1999). In young adulthood and midlife, social networks are more dynamic and plastic; their composition, on average, consists of more people from diverse areas of living, such as family, friends, and coworkers and involve the mix of both close relationships and weak ties. In old age, social networks typically have come to be what they are from a lifelong process of selective pruning (Carstensen et al., 1999) and may consist of fewer weak ties that could potentially fill the void of network members who pass away. Older adults place stronger value or get more from emotional goals and relationships (Charles, 2010).

According to the strength and vulnerability integration model, older adults are more adept at using thoughts and behaviors to avoid or mitigate exposure to stressful events (Charles, 2010). This is one general line of reasoning for why older adults are better able to overcome daily stressors as indexed by showing less emotional reactivity (Almeida, 2005; Blanchard-Fields, 2007). However, an additional assertion of the strength and vulnerability integration model is that in situations in which individuals experience high levels of distress, age differences that normally favor older adults will be attenuated (Charles, 2010). Repeated bereavements over several years of time can potentially qualify as instances of increased distress where these better strategies of overcoming stress are comprised, especially if these strategies involved recruiting network members to help overcome stressful events that arise. Different from daily negative events, which have the potential to be avoided or confronted more centrally, deaths in one’s network are outside of one’s own control. As a result of not having the ability to escape the situation, subjective well-being may be disproportionately affected in older adults, compared to younger and midlife adults. The roles and place of each network member and strategies for overcoming stress is a result of a lifelong, *active* process. Conversely, repeated bereavement in social networks is beyond one’s own control and with increases in losses in social network members being largely unavoidable, this can result in even stronger and sustained declines. These continued and increased negative experiences may lead to a spiraling of declines in subjective well-being.

For individuals in young adulthood, repeated bereavements may be considered a non-normative event (Hultsch & Plemons, 1979; Neugarten & Hagestad, 1976). It is not expected to lose social network members during this stage of adulthood and coupled with the transition to employment and familial transitions (i.e., marriage, child birth), could exacerbate the effects of repeated bereavement on subjective well-being. In contrast to individuals in young adulthood, those in midlife are involved in a complex interplay of work, family, and changing health (Lachman, 2004; Lachman, Teshale, & Agrigoroaei, 2015).

### Future Directions

Moving from examining singular to repeated major life stressors provides numerous strengths and opportunities in furthering our knowledge of how events that transpire (both bad and good) have the potential to shape the course of development. Repeated losses of network members were most detrimental to subjective well-being for individuals in young adulthood and old age. If we had only examined one event, we would likely have not observed any differences in change as observed by the nonsignificant effects for initial bereavement and adaptation. Events often do not happen in isolation, but can result in an avalanche effect. Recent research by Schilling and Diehl (2014) using daily diaries has documented that stressors experienced over the course of several days and weeks can pile-up, leading to more sustained declines in well-being, over and above that of concurrent daily stressors. Similarly, following spousal loss, surviving spouses have an increased likelihood for poor health and mortality (Stroebe, Schut, & Stroebe, 2007). Some events just by their very nature have the potential to happen more frequently, such as unemployment or the focus of the present study, losses in one’s social network. Individuals may be able to adapt to the first event, but as shown by Luhmann and Eid (2009), the repeated occurrence can take their toll over long periods of time (years).

Why such “small” effects of repeated bereavement on subjective well-being with the overall sample? One potential reason is the annual observations, which may be too sparse to detect large enough changes. As discussed by Infurna and Luthar (2018), annual assessments (or even 6-month assessments) may be too sparse and not sensitive enough to detect immediate changes in well-being (and other outcomes), and for how long changes are observed for. With assessments that are 1 year apart, it is more difficult to observe immediate changes and this has been a topic of focus with 3-month intervals being most sensitive to changes in subjective well-being (for discussion, see Frijters et al., 2011; Infurna et al., 2017; Uglanova & Staudinger, 2013). More closely spaced observations would also be instrumental in identifying mechanisms underlying changes in subjective well-being following repeated bereavement. For example, maintenance of well-being or adaptation following bereavement could be due to greater appreciation for life and meaning-making of the situation. Furthermore, there is heterogeneity in adaption following bereavement, suggesting that for some individuals they are able to bounce back more quickly whereas for others they may show stronger declines. This heterogeneity could be result in such small effects and ultimately be due to factors not assessed, such as coping resources and previous resources that are known to impact adaption following adversity (Carr, 2003; Sullivan & Infurna, in press).

A second avenue for future research in the study of repeated events is the focus of outcomes beyond that of subjective well-being, such as character strengths and virtues. Character strengths and virtues broadly encompass which factors help individuals facilitate a broader connection with all of humanity and empathetic concern for others (Clement & Bollinger, 2016; Peterson & Seligman, 2004). Character strengths and virtues allow for facilitating a broader perspective and connection with all of humanity and their key role for competence in everyday life (Emmons, 1999). Could there be growth following repeated bereavement (i.e., post-traumatic growth, see Jayawickreme & Blackie, 2014) or would this result in sustained declines in key outcomes pertaining to how individuals relate to others more broadly? This point dovetails with our earlier argument of the sparser assessment points and how these outcomes are not examined as frequently.

### Limitations

We note several limitations of our study. First, we did not find between-person differences in reactions to repeated bereavements. This could be due to the sparse assessment period, leading to the inability to detect between-person differences in changes in subjective well-being. As discussed earlier, future investigations with more closely spaced assessments promises to provide the opportunity to more closely examine whether and how life events have immediate impacts across pertinent outcomes. Second, we did not know the quality of the relationship between the individual and the close family member or friend who passed away. Degree of closeness and quality of social relationship is shown to moderate changes to bereavement (see Isherwood, King, & Luszcz, 2012) and is an important factor to consider in future investigations involving the effect of repeated bereavement. Along these lines, we did not have information on the role of the family member or friend in the social network. The loss of a person with a more central role (e.g., the sister who kept contact to everybody else in the family) could have a larger impact. Third, we were unable to differentiate the bereavements, such that the type of loss in one’s social network would likely differ across those in young adulthood, midlife, and old age. It could be that losses for younger adults were off time (e.g., parental) and for older adults more central to their network. Depending on who passed away, the cause, and their closeness in their social network composition, this can be a key reason for between-person differences in the extent of which change is observed. Future research is required to differentiate the types of losses experienced and the degree of closeness and how this may have a differential impact on subjective well-being. This could be one reason for the effect sizes being in the smaller range. Fourth, age and cohort in our analyses are confounded with our analyses making it difficult to parse age-period-cohort effects. Future research is required to disentangle the extent to which cohort differences are evident in repeated bereavements. Fifth, we are uncertain as to the individual’s experience of adversity prior to study entry. There is research documenting the extent to which lifetime adversity can impact resilience to other adversities encountered (Seery et al., 2010) and the present design did not allow for documenting adversity exposure across key domains and whether this plays a role in adaptation to repeated bereavement.

## Conclusion

Taken together, our study examined the effects of repeated bereavement on subjective well-being across the adult life span and whether there were differences across age. Our study contributes to and extends extant reports showing whether and how major life stressors have the potential to shape the course of development. We take our results to provide impetus to consider repeated events over the course of years and decades and their consequences for development in order to further examine the role of repeated life events on within-person changes over time. This promises to more thoroughly examining processes involved in the human capacity to overcome adversity.

## Conflict of Interest

None reported.
